# *N*-, *O*- and *S*-Heterocycles Synthesis in Deep Eutectic Solvents

**DOI:** 10.3390/molecules28083459

**Published:** 2023-04-14

**Authors:** Serena Perrone, Francesco Messa, Luigino Troisi, Antonio Salomone

**Affiliations:** 1 Dipartimento di Scienze e Tecnologie Biologiche ed Ambientali, Università del Salento, Prov.le Lecce-Monteroni, I-73100 Lecce, Italy; 2 Dipartimento di Chimica, Consorzio C.I.N.M.P.I.S., Università degli Studi di Bari “Aldo Moro”, Via E. Orabona 4, I-70125 Bari, Italy

**Keywords:** heterocyclic synthesis, deep eutectic solvents, H-bond catalysis

## Abstract

The synthesis of heterocycles is a fundamental area of organic chemistry that offers enormous potential for the discovery of new products with important applications in our daily life such as pharmaceuticals, agrochemicals, flavors, dyes, and, more generally, engineered materials with innovative properties. As heterocyclic compounds find application across multiple industries and are prepared in very large quantities, the development of sustainable approaches for their synthesis has become a crucial objective for contemporary green chemistry committed to reducing the environmental impact of chemical processes. In this context, the present review focuses on the recent methodologies aimed at preparing *N*-, *O*- and *S*-heterocyclic compounds in Deep Eutectic Solvents, a new class of ionic solvents that are non-volatile, non-toxic, easy to prepare, easy to recycle, and can be obtained from renewable sources. Emphasis has been placed on those processes that prioritize the recycling of catalyst and solvent, as they offer the dual benefit of promoting synthetic efficiency while demonstrating environmental responsibility.

## 1. Introduction

Heterocycles are very useful compounds in a breadth of fields including chemical synthesis and materials. Moreover, many compounds exhibiting significant biological and pharmacological activities are characterized by a heterocyclic core in their structure [1].

Over the past decades, as a result of widespread environmental issues, more attention has been paid to the development of new strategies for heterocycle synthesis, characterized by more green and eco-friendly conditions, particularly to avoid the use of toxic and volatile organic solvents. In recent years, deep eutectic solvents (DESs) have received considerable attention in organic syntheses as a green and sustainable alternative to volatile organic solvents and conventional ionic liquids (ILs) [2,3]. Indeed, as one of the green chemistry principles [4], the utilization of DESs in the preparation of heterocycles is gaining more and more attention.

First reported by Abbott in 2003 [5], DESs are defined as a mixture of at least two components that are capable of forming a eutectic mixture with a melting point lower than each component individually. Some of these mixtures could show a glass transition temperature point rather than a strict melting point and, therefore, they are also known as low transition temperature mixtures (LTTM) [3,6].

Today, DESs are classified into five main types based on their composition [3,7]. Among these, type III DESs are a popular choice due to their high level of environmental sustainability, as they often utilize components derived from natural sources. They are formed by hydrogen bond acceptors (HBA), such as quaternary ammonium salts, and hydrogen bond donors (HBD) in different molar ratios. The HBDs employed are often urea or its derivatives, glycerol, other polyols, carbohydrates, carboxylic acids, etc. [6]. Because of their low toxicity, easy preparation, chemical stability, non-volatility, biodegradability, non-flammability, and recyclability, the use of DESs as green media in the synthesis of organic compounds [3,6], including heterocycles [8], has greatly increased over the last decade.

As described in this Review, generally the eutectic mixture plays an active role in the chemical transformation, as DES not only acts as an eco-friendly solvent but also could be used as a catalyst, due to the strong network of hydrogen bonds between the reagents (or reaction intermediates) and the DES components.

Additionally, DESs offer a range of benefits that make them an attractive choice for various applications. These include simple work-up procedures, short reaction times, high yields of the desired products, mild reaction conditions, affordability, availability, and the potential for reusability of the eutectic mixture, all of which contribute to the effectiveness of the reported methodologies.

**Figure 1:**
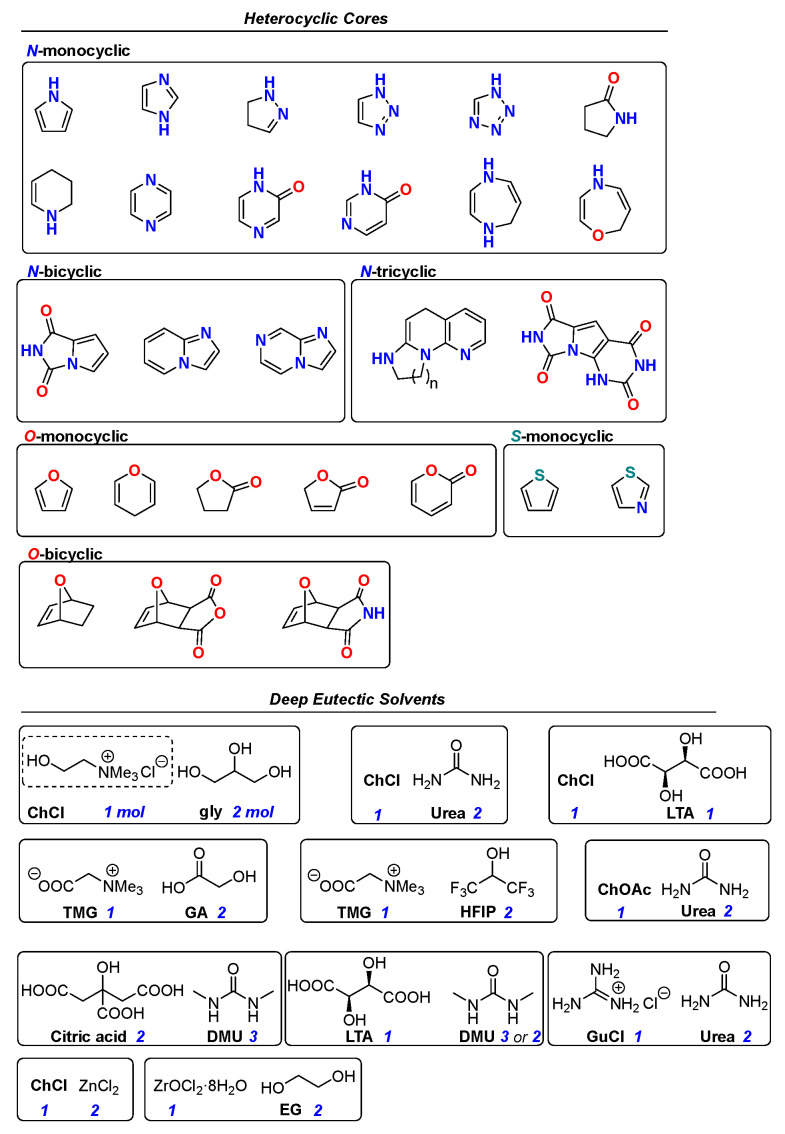
Molecular structures of heterocyclic cores and deep eutectic solvents (with molar ratio) reviewed. ChCl = Choline chloride; gly = glycerol; LTA = *L*-(+)-tartaric acid; TMG = trimethylg lycine or betaine; GA = glycolic acid; HFIP = hexafluoro i-propanol; ChOAc = choline acetate; DMU = *N*,*N*′-dimethylurea; GuCl = guanidinium chloride; EG = ethylene glycol. The molar ratio of DES components is shown in blue.

This Review, providing contributions on the preparation of heterocyclic compounds in a wide range of deep eutectic solvents, aims to give an overview of the advances, from the year 2014 to date, in the field of sustainable synthesis of heterocycles. In Figure 1 are reported the molecular structures of heterocyclic cores are reviewed as well as the eutectic mixture used. This manuscript is organized into further three sections, according to the type of heterocycles: *N*-, *O*- and *S*-heterocycles, and miscellaneous examples. Each section has been divided into sub-sections according to the heterocycle size (five-, six- and/or seven-membered ring).

## 2. Preparation of *N*-Heterocycles in DES Mixtures

### 2.1. Synthesis of Five-Membered N-Heterocycles

Among the different heterocyclic compounds, imidazole-containing moiety is widely known due to its broad range of chemical and biological properties [9].

The 2-amminoimidazole scaffold, for example, represents an important structural motif present in a wide range of bioactive compounds [10,11,12,13].

**Scheme 1:**
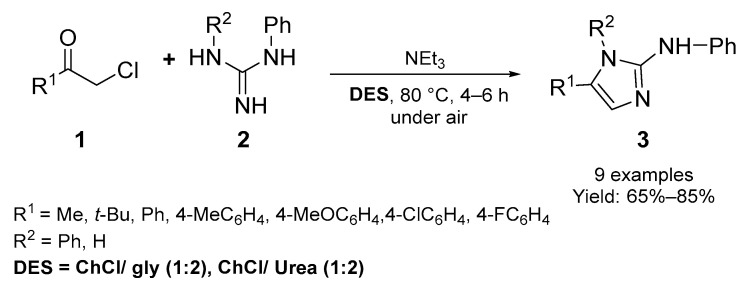
Preparation of 2-amminoimidazole *N*-heterocycles **3** in ChCl-based DESs.

In 2016, Capua and co-workers studied the reactivity of variously substituted α-chloroketones **1** with guanidine derivatives **2**, employing choline chloride (ChCl)-based DESs [ChCl/glycerol (gly) and ChCl/urea] as environmentally friendly and safe reaction media (Scheme 1) [14]. Both di- and monophenyl guanidine **2** have been shown to be good partners with α-chloroketones to successfully afford, through a cyclodehydration reaction, 2-amminoimidazoles **3**, a class of biologically relevant molecules [10,11,12,13], obtained in moderate to good yield (65–85%, Scheme 1).

Moreover, it must be emphasized that, in some cases, the purification of the synthesized imidazoles took place avoiding the use of the typical column chromatographic techniques and toxic and hazardous volatile organic solvents. In particular, triaryl-substituted 2-amminoimidazoles prepared in ChCl/urea as the eutectic mixture could be easily isolated by means of a simple workup procedure involving filtration and crystallization processes, also allowing the DES mixture to be recovered and recycled for three cycles [14].

**Scheme 2:**
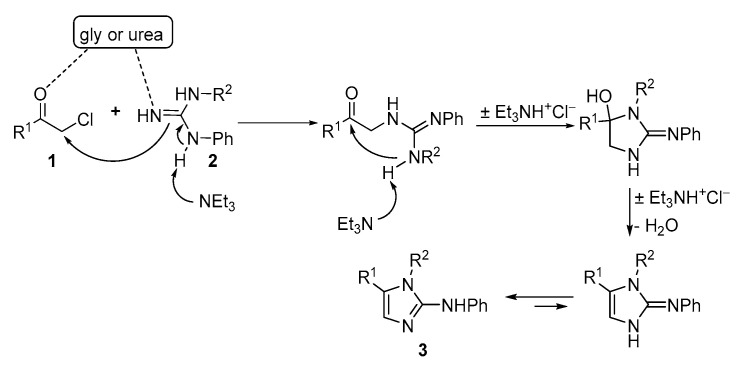
The hypothesized mechanism, involving hydrogen bond catalysis promoted by DES components, for the formation of 2-aminoimidazole derivatives **3** in ChCl-based DESs.

A hydrogen bond catalysis, promoted by DES components, such as glycerol and urea, was postulated to be involved in the activation of the carbonyl moiety and the guanidine derivative, promoting the formation of 2-amminoimidazoles in DES mixtures, Scheme 2 [14].

**Scheme 3:**
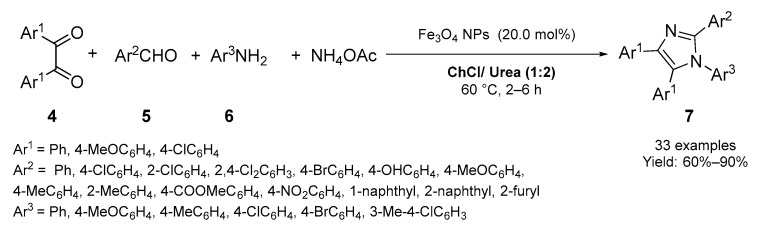
Synthesis of tetrasubstituted imidazoles **7** with DES-stabilized Fe_3_O_4_ NPs.

Furthermore, a one-pot multi-component reaction was developed by Manochehri and coworkers for the synthesis of tetrasubstituted imidazoles, in ChCl/urea as DES [15]. Particularly, in the presence of DES-stabilized iron oxide nanoparticles (Fe_3_O_4_ NPs) as a catalyst, the four-component reaction of a 1,2-dione (**4**), an aromatic aldehyde (**5**), a primary aromatic amine (**6**) and ammonium acetate afforded, at 60 °C, a range of imidazole derivatives **7** in moderate to excellent yields (60–90%, Scheme 3). For the ChCl-based DES an involvement in hydrogen bond catalysis, as well as in the stabilization of Fe_3_O_4_ NPs, was supposed [15].

**Scheme 4:**
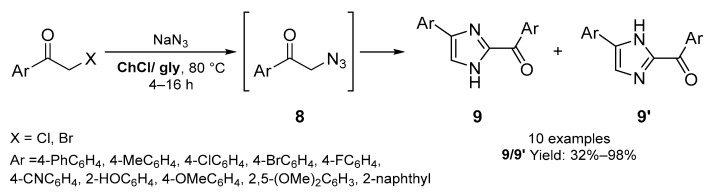
Synthesis of 2-aroyl-(4 or 5)-aryl-(1*H*)-imidazoles **9/9′** in ChCl/gly DES.

In 2020, Vitale et al. reported the synthesis of functionalized imidazoles **9/9′** in ChCl/gly as DES (Scheme 4). Specifically, it was shown that chloro- and bromomethyl ketones, when treated with NaN_3_ at 80 °C, for 4–16 h, could be easily converted into a mixture of two tautomeric imidazoles [2-aroyl-(4 or 5)-aryl-(1*H*)-imidazoles **9/9′**], through the formation the arylacyl azide **8** as a key intermediate [16]. This method allowed the synthesis of a range of heterocycles **9/9′** in yields ranging from 32% to 98% (Scheme 4). Hydrogen bond catalysis promoted by DES components could play a central role in favoring the process. It is noteworthy how the authors, using the same procedure, were able to develop a regiodivergent synthesis. Indeed, by employing ChCl/urea (1:2 mol/mol) as a non-innocent reaction medium and by modulating the reaction temperature (25 or 80 °C), as well as the presence or absence of bases (Et_3_N), the conversion of the arylacyl azide intermediate **8** into pyrimidine derivatives, was observed [16].

**Scheme 5:**
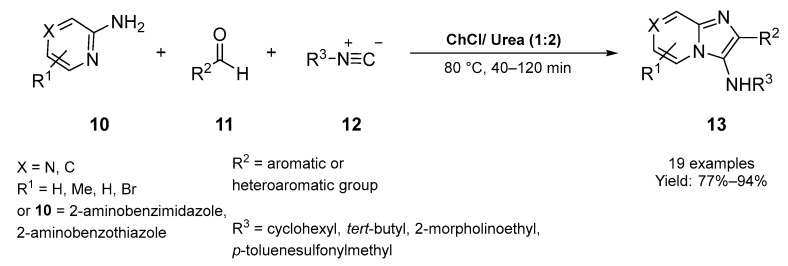
Synthesis of 3-aminoimidazo-fused heterocycles **13** using ChCl/urea as DES.

The eutectic mixture ChCl/urea was found to be both an eco-friendly and biodegradable solvent and an organo-catalyst able to promote the multicomponent synthesis of variously substituted 3-aminoimidazo-fused heterocycles **13** via Groebke–Blackburn–Bienayme (GBB) reaction, Scheme 5 [17]. Imidazole derivatives fused with a heterocycle, such as a pyridine or pyrimidine ring, are bioactive molecules showing antiviral [18,19] and antifungal [20] activities. Particularly, the GBB three-component reaction, involving a heteroaromatic primary amine **10**, an aromatic or heteroaromatic aldehyde **11,** and an aliphatic isocyanide **12**, led to the heterocycles **13** in high yield (77–94%) and short reaction times (40–120 min), under metal-free catalysis (Scheme 5) [17]. Furthermore, ChCl/urea DES could be reused for consecutive runs without a considerable loss in the product yield.

**Scheme 6:**
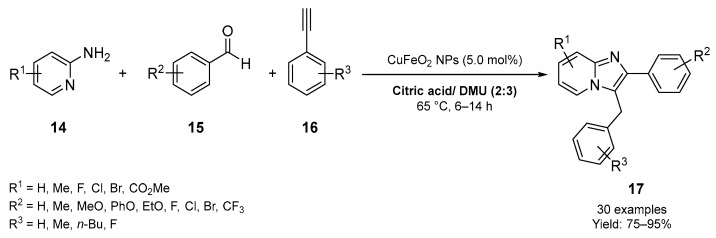
Synthesis of imidazo[1,2-*a*]pyridine derivatives **17** through a tree-component reaction catalyzed by CuFeO_2_ in citric acid/DMU.

Other synthetic strategies to obtain bicyclic imidazo-fused heterocycles, based on the successful combination of metal-based nanoparticles (NPs) and eutectic mixtures [21], have also been described by Lu and co-workers. Specifically, imidazo[1,2-*a*]pyridine derivatives, were prepared through a three-component reaction, involving 2-aminopyridines **14**, aldehydes **15,** and terminal alkynes **16** [22]. Superparamagnetic CuFeO_2_ NPs were used as catalytic species, in the melting mixture citric acid/dimethylurea (DMU) (2:3 mol/mol), obtaining the desired imidazo[1,2-*a*]pyridine derivatives **17** in good to excellent yields (75–95%), and with a ranged substrate scope (Scheme 6). The recyclability of both catalyst and solvent was also investigated: it was possible to repeat six successive cycles reusing the melting mixture and the catalyst, without any significant drop in the reaction yield.

**Scheme 7:**
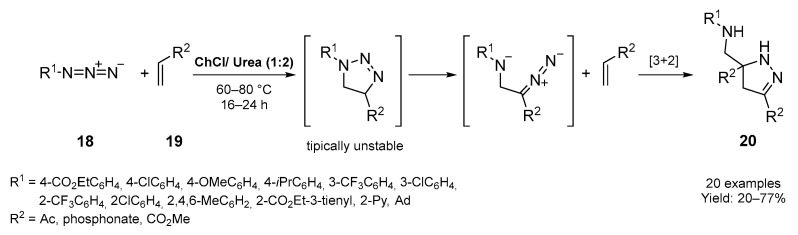
Synthesis of 2-pyrazolines **20** through a [3+2] cycloaddition reaction between electro-deficient azides and alkenes in ChCl/urea DES.

In 2020, Sebest et al. reported the synthesis of 2-pyrazolines **20**, a class of bioactive heterocycles [23], via a [3+2] cycloaddition reaction between electron-deficient azides **18** and alkenes **19,** through the formation of a transient 1,4-disubstituted triazoline intermediate (Scheme 7) [24]. The eutectic mixture composed of ChCl/urea was found to be the best reaction medium, both significantly reducing the amount of volatile organic solvents employed in the process and substituting the base that promotes the formation of 2-pyrazolines. Furthermore, the low vapor pressure of DES made it possible to carry out the reaction at higher temperatures compared to the previously reported solvents (typically toluene or methanol), greatly reducing the reaction time. It should be emphasized that, depending on the electron-poor alkene derivatives employed in the reaction, different heterocycles, such as aziridines, triazoles, or triazolines, could be formed as reaction products [24].

**Scheme 8:**
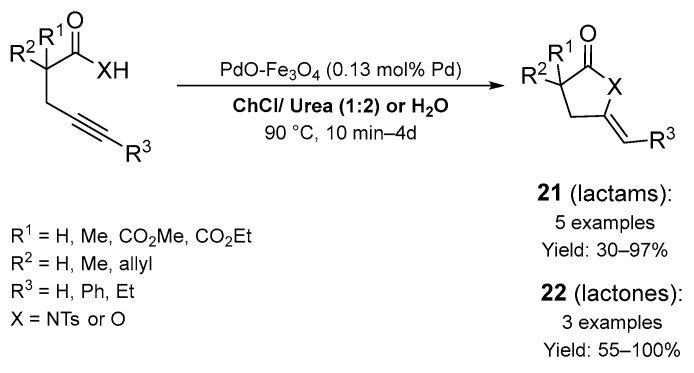
Cycloisomerization of alkynyl sulfonylimides and alkynoic acid derivatives by PdO–Fe_3_O_4_ in sustainable solvents.

Furthermore, highly substituted five-membered lactams could be prepared in deep eutectic solvents. Recently, a metal-catalyzed cycloisomerization process of alkynyl sulfonylimides or alkynoic acids to obtain lactams **21** or lactones **22**, respectively, was reported in sustainable solvents [25]. Particularly, Saavedra and co-workers described that a heterogeneous palladium(II) catalyst supported on magnetite (PdO-Fe_3_O_4_) can be efficiently and selectively employed for the cycloisomerization of alkynoic acids and their *N*-tosyl derivatives, both in aqueous medium and in ChCl/urea eutectic mixture, working with low catalyst loading, under aerobic conditions, at 90 °C and in the absence of co-catalysts (Scheme 8). Moreover, the recoverability of the heterogeneous palladium(II) oxide impregnated on magnetite was evaluated; indeed, the catalytic system in water could be reused for up to four consecutive cycles, without any decrease in catalytic activity and selectivity [25].

Pyrrole and its derivatives represent a valuable class of *N*-heterocycles widely found in biologically active natural compounds, pharmaceuticals, ligands, and synthetic building blocks [26].

**Scheme 9:**
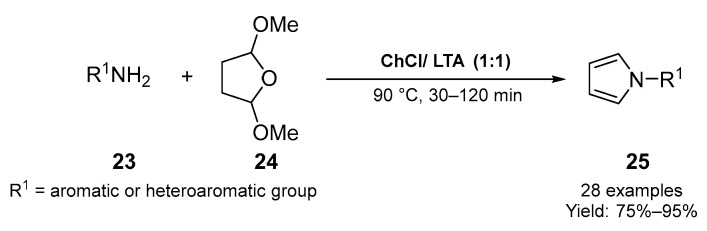
Preparation of pyrroles derivatives **25** in ChCl-based DES.

In 2014, Wang et al. showed that a DES composed of ChCl/*L*-(+)-tartaric acid (LTA) could be both an environmentally benign and reusable reaction medium and catalyst for the preparation of *N*-substituted pyrroles **25** through a Clauson-Kaas reaction, Scheme 9 [27]. The reaction between aromatic or heteroaromatic primary amines, bearing electron-donating or withdrawing groups, and 2,5-dimethoxytetrahydrofuran provided structurally different *N*-substituted pyrroles **25**, under mild conditions and short reaction time. The electron properties of the substituent of the amine had little influence on the reaction trend and the heterocycles **25** were obtained in good to excellent yields (75–95%, Scheme 9). Moreover, the eutectic mixture ChCl/LTA was recycled five times and the results of five consecutive runs showed almost consistent yields [27].

**Scheme 10:**
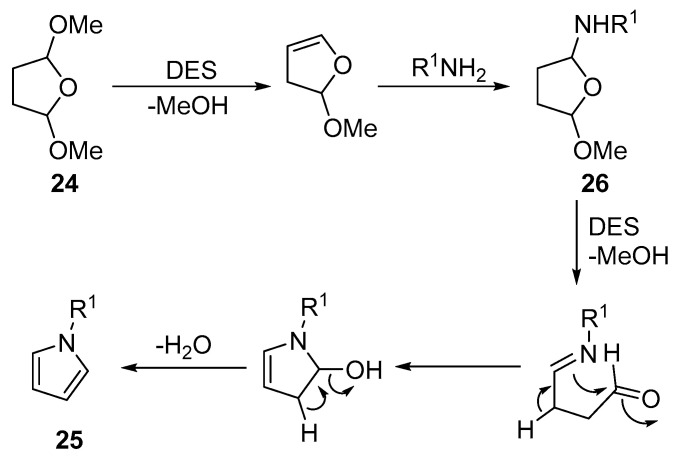
Suggested reaction mechanism for the formation of *N-*substituted pyrroles **25** in DES.

A reaction mechanism for the formation of *N*-substituted pyrroles has been proposed in which the acidity of the DES could play a key role to promote the expulsion of MeOH from the tetrahydrofuran and 4,5-dihydrofuran derivatives **24** and **26**, Scheme 10 [27].

**Scheme 11:**
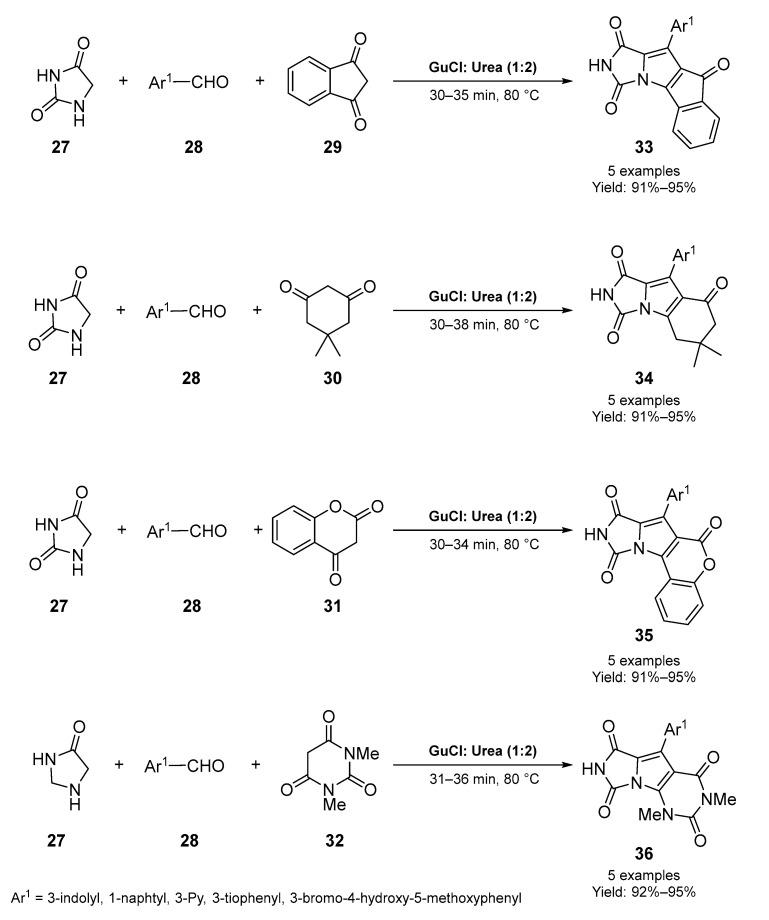
Synthesis of imidazole-pyrrole derivatives **33**–**36** in GuCl/urea DES.

Furthermore, using a DES base on guanidinium chloride (GuCl) and urea, a great variety of pyrrole-imidazole derivatives, such as indenopyrroloimidazoles **33**, imidazoindoles **34**, chromenopyrroloimidazoles **35**, imidazopyrrolopyrimidines **36**, were synthesized, in a short reaction time (30–38 min) and under mild conditions [28]. As shown in Scheme 11, this three-component domino reaction, involving hydantoin **27**, an aromatic or heteroaromatic aldehyde **28,** and a ketone compound (**29**–**32**), afforded the heterocyclic structures **33**–**36** in excellent yields (91–95%). Moreover, the eutectic mixture GuCl/urea was easily recovered and reused six times without an appreciable loss of reaction yield. A dual role of the DES, as a sustainable reaction medium and as a promoter of the reaction, was hypothesized. Particularly, the DES, through the formation of noncovalent interactions between its components and the reaction reagents and intermediates, could increase the efficiency of the reaction and thus facilitate the process [28].

Among the five-membered *N*-heterocycles, the triazole scaffold has become very attractive because due to its wide range of bioactivities [29,30].

**Scheme 12:**
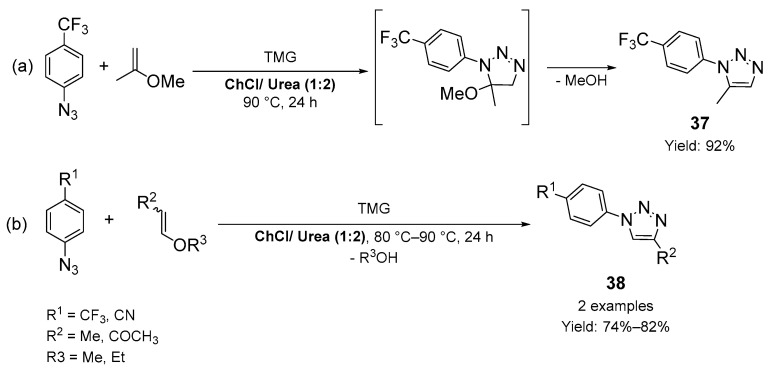
One-pot synthesis of 1,5- and 1,4-disubstituted 1,2,3-triazoles **37** and **38**, respectively, in ChCl/urea eutectic mixture.

In 2019, Sebest and coworkers reported the first example of a metal-free synthesis of 1,5-disubstituted 1,2,3-triazoles **37** (Scheme 12a) and 1,4-disubstituted 1,2,3-triazoles **38** (Scheme 12b), via one-pot azide-alkene cycloaddition-elimination sequence in deep eutectic solvents [31]. The combination ChCl/urea (as DES) and tetramethylguanidine (TMG, as a base) resulted in the best one to prepare the triazole rings, Scheme 12. However, the reaction showed a limited substrate scope, and also with the use of alternative leaving groups on the alkene (i.e., silyl enol ethers and triflate enol ethers), the desired disubstituted 1,2,3-triazole derivatives were obtained in low yields (0–24%) in DES.

**Scheme 13:**
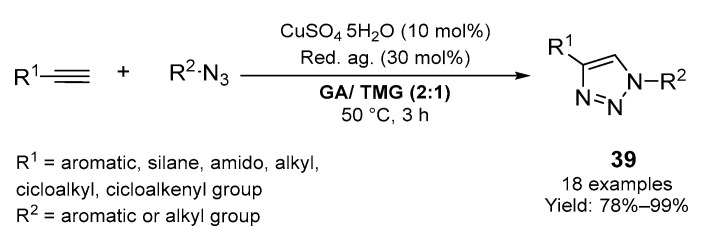
Cycloaddition reaction between terminal alkynes and organic azides to obtain 1,2,3-triazoles **39** in GA/TMG eutectic mixture.

In 2021, Giofrè’s and Tiecco’s research groups showed a copper-catalyzed 1,3-dipolar cycloaddition reaction between terminal alkynes and organic azides, performed in natural DESs, affording variously substituted 1,2,3-triazoles **39** [32]. The reaction, carried out in base-free conditions, proceeded in two different eutectic mixtures, glycolic acid/trimethylglycine (GA/TMG, 2:1 mol/mol) and ChCl/ascorbic acid (ChCl/Asc, 2:1 mol/mol), although GA/TMG showed to be the best green solvent to produce 1,2,3-triazole derivatives **39** in good to quantitative yields (78–99%), from a variety of alkynes and azides linked to aromatic or alkyl groups (Scheme 13). On the contrary, ChCl/Asc DES, due to its reducing properties, avoided the use of reducing agents in the reaction mixture, albeit the reaction led to the formation of triazole derivatives in good yield (75–97%) only with terminal aromatic alkynes. An “active” role of the DES was suggested, assuming a hydrogen bond catalysis and an involvement in the stabilization of catalytically active copper intermediates. Moreover, GA/TMG eutectic mixture was found to be easily recycled, keeping the reaction yield almost unchanged for three consecutive runs [32].

**Scheme 14:**
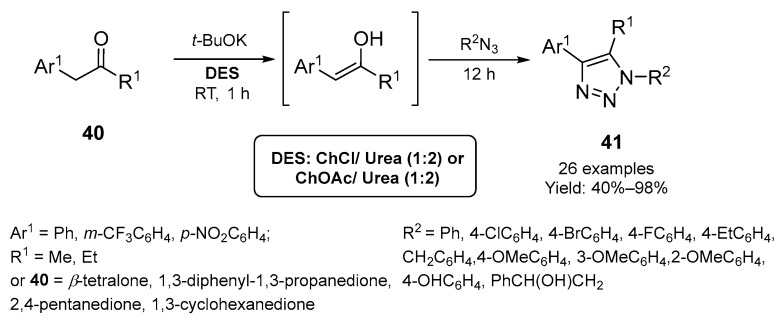
Synthesis of functionalized 1,2,3-triazole derivatives **41** in eutectic mixtures.

Recently, Cicco and coworkers described a regioselective synthesis of functionalized 1,2,3-triazole derivatives **41**, through a 1,3-dipolar cycloaddition reaction between enolates of alkanones **40** and azides, in environmentally benign urea-based eutectic mixtures [ChCl/urea and choline acetate (ChOAc)/urea], Scheme 14 [33]. This metal-free protocol, performed under mild conditions such as room temperature and aerobic conditions, showed a broad substrate scope, affording the desired triazoles in 40–98% yields, overall, up to 13 h, Scheme 14. The eutectic mixture ChOAc/urea was also recycled for four runs, showing a decrease in the product yield from 98% (first cycle) to 66% (fourth cycle). Moreover, one-pot cycloaddition/reduction processes were successfully performed in ChCl/urea DES leading to functionalized triazoles with pharmacological properties [33].

Moreover, the tetrazole ring has been prepared in DES mixtures. The tetrazole nucleus is an important five-membered heterocycle, widely found in many natural products, and in several drugs and drug candidates [34].

**Scheme 15:**
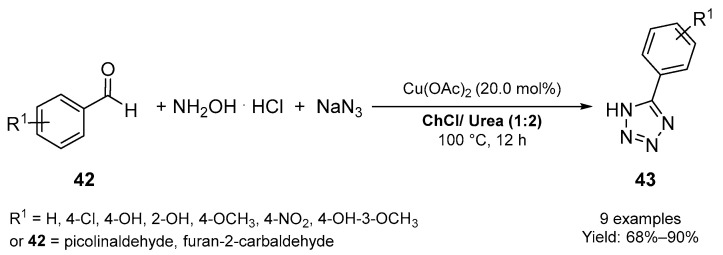
Synthesis of 5-substituted-1*H*-tetrazole derivatives (**43**) in ChCl/urea DES.

In 2019, Xiong and coworkers prepared a series of 5-substituted-1*H*-tetrazole derivatives (**43**) through a one-pot cascade reaction involving an aryl aldehyde, hydroxylamine hydrochloride, and sodium azide [35]. The reaction, affording tetrazoles **43** in moderate to excellent yields (68–90%), was performed in the presence of Cu(OAc)_2_ as the catalyst and used ChCl/urea mixture as an eco-friendly solvent (Scheme 15). According to the authors, the system DES-Cu(OAc)_2_ could promote the [3+2] cycloaddition between sodium azide and the C=N bond of benzaldehyde oxime generated in situ, affording, after H_2_O elimination, the desired tetrazole product [35].

**Scheme 16:**
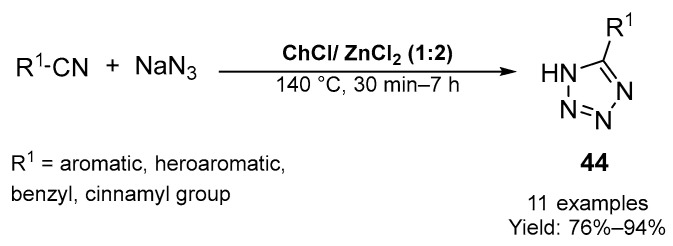
Synthesis of 5-substituted-1*H*-tetrazole derivatives (**44**) in ChCl/ZnCl_2_ DES.

To obtain 5-substituted-tetrazole derivatives, a [3+2] cycloaddition reaction was also performed between organic nitriles and sodium azide, employing a type I DES, namely ChCl/ZnCl_2_ [36]. The eutectic mixture was found to play a dual role both as reaction medium and catalyst, and it could be recycled up to four consecutive runs. Aromatic nitriles bearing both electron-donating and withdrawing groups, benzyl nitriles, as well as (*E*)-cinnamonitrile were smoothly converted into the desired heterocycles **44** within 0.5–7 h reaction time, at 140 °C, with good to excellent yields (76–94%), Scheme 16 [36].

### 2.2. Synthesis of Six- and Seven- Membered N-Heterocycles

**Scheme 17:**
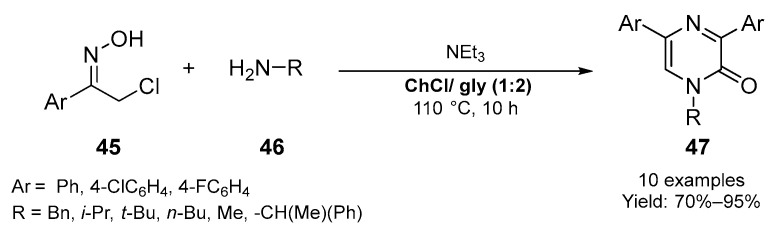
Synthesis of 1-alkyl-3,5-aryl trisubstituted 2(1*H*)-pyrazinones **47** in ChCl/gly DES.

1-Alkyl-3,5-aryl trisubstituted 2(1*H*)-pyrazinones **47**, biologically valuable compounds as peptidomimetic scaffolds [37,38,39], were successfully prepared employing the DES ChCl/gly as a green medium for the reaction (Scheme 17) [40]. Specifically, these nitrogen-containing heterocycles, obtained with good to excellent yields (70–95%), were synthesized through a simple coupling reaction between unsaturated halides, such as aromatic *α-*chloro oximes **45**, and aliphatic primary amines (**46**), after a reaction time of 10 h and a temperature of 100 °C (Scheme 17). The procedure worked efficiently also using sterically demanding amines (i.e., *tert*-butylamine and 1-phenylethan-1-amine) as nucleophiles, affording the corresponding pyrazinone derivative **47** in excellent yields (91–95%). Moreover, the DES mixture ChCl/gly was easily recovered and reused for three consecutive experiments, without a significant decrease in the chemical yield [40].

**Scheme 18:**
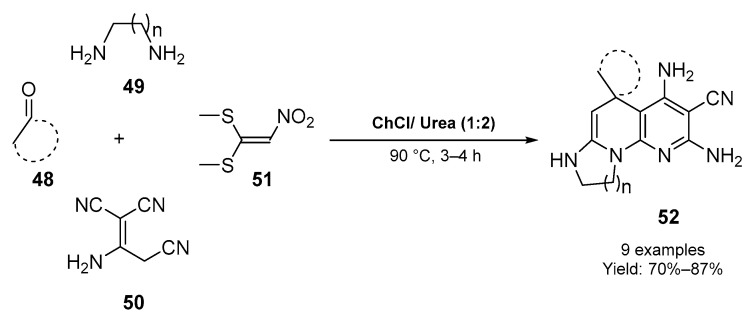
Preparation of 1,8-naphthyridine derivatives **52** via a domino four-component reaction in ChCl/urea DES.

1,8-Naphthyridine and its derivatives are *N*-heterocycles with great chemical and biological significance. Indeed, this scaffold is present in many products that possess numerous biological activities [41,42,43]. In 2016, Shaabani and co-workers reported an environmentally friendly approach for the preparation of poly-functionalized fused naphthyridine derivatives **52**, via a domino four-components reaction in the eutectic mixture ChCl/urea (Scheme 18) [44]. Particularly, fully substituted naphthyridines **52** were synthesized by reacting a diamine **49** (i.e., ethylenediamine, propanediamine, and 1,2-diaminocyclohexane), 1,1-bis(methylthio)-2-nitroethylene **51**, 2-aminoprop-1-ene-1,1,3-tricarbonitrile **50**, and a carbonyl compound **48**, such as benzaldehyde derivatives, isatin, ninhydrin, and naphthyl-2-carbaldehyde, in the DES ChCl/urea, under mild conditions and without the addition of an external catalyst (Scheme 18). It was proposed that the DES could have a dual role in the reaction, both as a green reaction medium and as a catalyst for the process. Indeed, the urea component of the DES could activate the carbonyl cyano groups of the reagents via hydrogen bond formation [44].

**Scheme 19:**
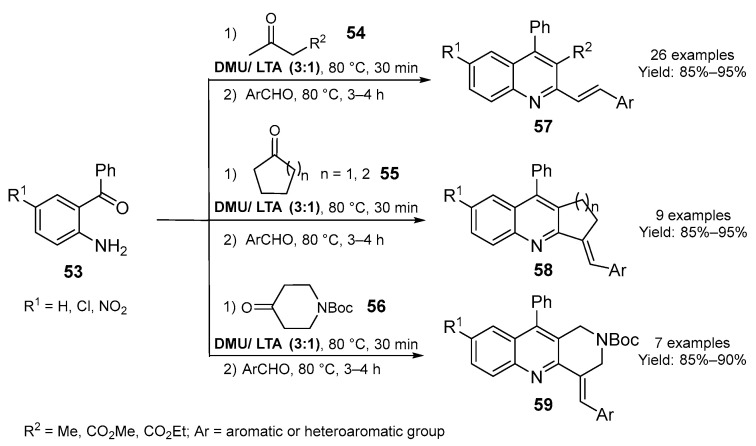
Synthesis of 2-styrylquinolines **57–59** in DMU/LTA DES.

The quinazoline ring, a valuable scaffold found in many biologically active compounds [45], has been prepared in eutectic mixtures. In 2022, Kashinath and coworkers described a two-step one-pot synthesis of 2-styrylquinolines **57**–**59** with interesting optical properties (Scheme 19) performed in a DES [46]. Particularly, the benzophenone derivative **53** reacted with a carbonyl compound (**54**–**55**) or with the Boc-protected piperidin-4-one **56**, to produce, in situ, a quinoline derivative that, after the reaction with suitable aromatic or heteroaromatic aldehydes, afforded the corresponding 2-styrylquinoline derivatives **57**–**59** (Scheme 19). The synthetic method worked under metal-free conditions, at 80 °C, using the eutectic mixture composed of 1,3-dimethyl urea (1,3-DMU) and LTA, in a 3:1 ratio, as the reaction medium. The reaction proceeded via a Friedlander annulation followed by Knoevenagel condensation (sp^3^ C–H activation) to give the products **57**–**59** in good to excellent yields (85–95%, Scheme 19). About the role of DES, it was suggested, based on density functional theory (DFT) calculations, that it could be involved in sp^3^ C–H activation, by promoting the enolization of the carbonyl compound (for Friedlander annulation) and the formation of enamine [46].

**Scheme 20:**
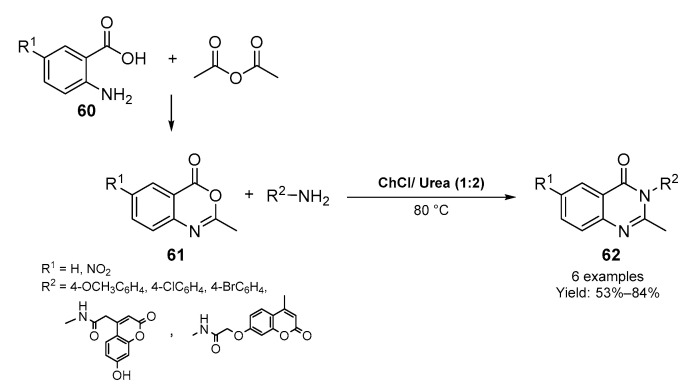
Preparation of quinazolinone derivatives **62** in ChCl/urea DES.

Moreover, 3-substituted-quinazolin-4(*3H*)-one derivatives were prepared by Komar et al. [47], using ChCl/urea eutectic solvent, in a two-step cyclization reaction. Quinazolinones are a class of six-membered heterocycles exhibiting a range of significant pharmacological properties, including antitumor activities [48]. In particular, for the synthesis of 2-methyl-3-substituted-quinazolin-4(*3H*)-ones **62**, at first, the preparation of benzoxazinone **61**, as an intermediate, starting from anthranilic acid **60** and acetic anhydride, was carried out. Afterwards, benzoxazinone **61** and a primary amine were added to the ChCl-based DES, affording, under heating at 80 °C, the corresponding benzo-fused derivatives **62** in 53–84% yield (Scheme 20). A dual role of the DES, as both the green medium and catalyst, through the formation of hydrogen bonds with the reactants and reaction intermediates, was hypothesized [47].

**Scheme 21:**
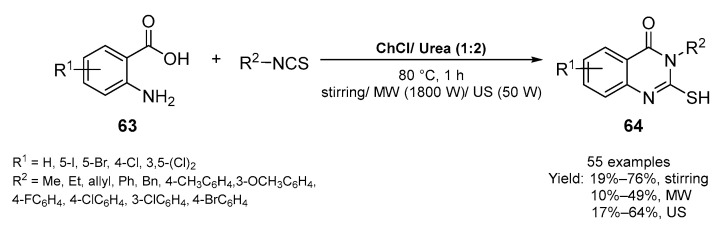
Preparation of substituted 2-mercaptoquinazolin-4(3*H*)-ones **64** in ChCl/urea DES.

In 2022, the same authors, starting from aliphatic or aromatic isothiocyanates and variously substituted anthranilic acids (**63**), were also able to synthetize in DES 2-mercaptoquinazolin-4(3*H*)-one derivatives of **64**, Scheme 21 [49]. Twenty different ChCl-based eutectic mixtures were screened, showing the combination ChCl/urea 1:2 mol/mol as the most effective to perform the reaction. In addition, a comparison of product yields obtained with conventional stirring, microwave-assisted or ultrasound-assisted synthesis was carried out. In the reaction performed at 80 °C for 1 h, the use of stirring or ultrasounds led to higher yields of the heterocycles **64** (17–76%), while microwave-induced synthesis showed lower results (**64** yields: 10–49%), Scheme 21. Moreover, the recyclability of the DES was examined showing that the reusability of the eutectic mixture was good for four recycles, without any appreciable reduction in the product yield [49].

**Scheme 22:**
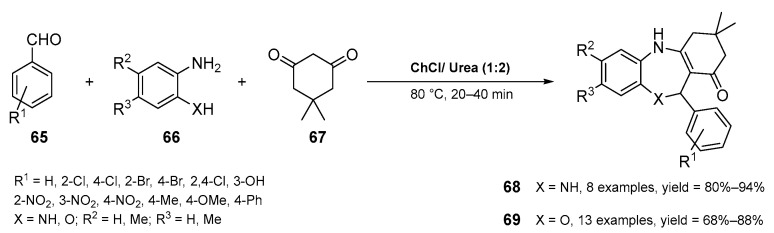
Preparation of tricyclic 1,4-benzodiazepines **68** and 1,4-benzoxazepines **69** via a three-components reaction in ChCl/urea DES.

The same eutectic mixture ChCl/urea has been applied both as a reaction medium and as a promoter for the synthesis of benzo-fused seven-membered *N-*heterocyclic systems such as tricyclic 1,4-benzodiazepines **68** and 1,4-benzoxazepines **69** (Scheme 22) [50]. Diazepines and their benzo-fused derivatives have attracted considerable interest in organic synthetic chemistry, especially because of their pharmacological significance [51,52]. These benzo-fused seven-membered heterocycles **68**–**69** were prepared in a ChCl-based DES at 80 °C through a one-pot, a multicomponent reaction involving (1) a benzaldehyde derivative **65**, containing electron-donating or electron-withdrawing groups, (2) *o*-phenylenediamine or 2-aminophenol **66** and (3) dimedone **67** (Scheme 22) [50]. The benzodiazepine derivatives **68** were obtained in higher yields (80–94%) and in shorter reaction times (20–30 min) compared to the benzoxazepines **69** (68–88%, 30–40 min), both heterocycles without the use of additional metal or acid catalysts. Indeed, DES components, via hydrogen binding, could be able to activate carbonyl and imine groups of reagents and reaction intermediates, promoting the multicomponent reaction. The reusability of the eutectic mixture was evaluated too, obtaining excellent results for four consecutive reaction runs [50].

**Scheme 23:**
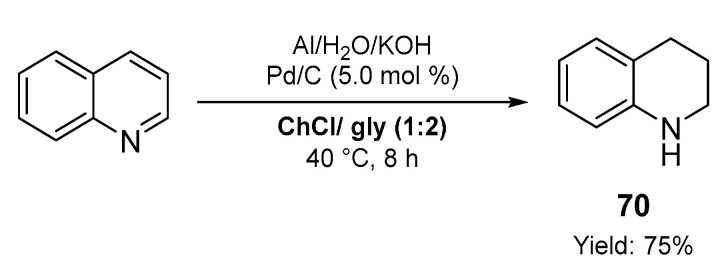
Aluminum-promoted reduction of quinoline in ChCl/gly as DES to provide 1,2,3,4-tetrahydroquinoline scaffold **70**.

Reductive strategies can be employed for the preparation of *N*-heterocycles. Hydrogenation reactions, and more generally the reductions, providing a direct route for the formation of C–H, N–H, and O–H bonds, are among the most used transformations in the manufacture of pharmaceuticals, bulk, and fine chemicals [53,54,55]. Our research group has recently reviewed recent advances in the field of reducing synthetic strategies with a low environmental impact, performed in green solvents derived from renewable sources [56]. Such sustainable reductive methodologies were also used to prepare reduced six-membered *N*-heterocycles**.** As a part of our ongoing interest in metal-catalyzed reactions [57,58,59,60,61] and sustainable synthetic methodologies [14,40,62,63], a Pd/C-catalyzed reductive process, involving in situ generation of H_2_, from Al powder and basic water, was recently developed in the bio-based ChCl/gly eutectic mixture for the hydrogenation of a variety of organic compounds and for the de-aromatization of heteroaromatic rings such as quinoline (Scheme 23) [64]. Particularly, the quinoline heterocycle could be smoothly reduced in a good yield of 75%, affording the 1,2,3,4-tetrahydroquinoline **70**, a valuable scaffold for the preparation of bio-active compounds (Scheme 23) [65]. Moreover, the role of DES in the hydrogenation process has been postulated: the eutectic mixture, besides being a sustainable medium, also takes part in the activation of aluminum particles by removing the Al_2_O_3_ protective layer from the metal surface, as well as makes a fairly safe process, due to its the insignificant volatility and the low thermal conductivity [64].

**Scheme 24:**
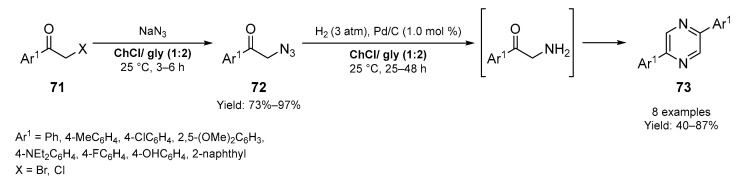
Synthesis of pyrazines **73** in the DES ChCl/gly.

The sustainable and eco-friendly eutectic mixture ChCl/gly proved to be an effective medium also to perform the synthesis of valuable six-membered *N*-heterocycles, symmetrical 2,5-diarylpyrazines **73** [66]. Pyrazine derivatives are a class of cyclic compounds with a wide range of applications in many areas, including the pharmacological field [67] as well as the coordination chemistry [68]. Compounds **73** were easily prepared from aryl azides (**72**), in yield up to 87%, via a catalytic hydrogenation process involving the heterogenous catalyst Pd/C (1.0 mol%) and 3 atm pressure of H_2_, Scheme 24. Aryl azides **72** were synthesized in the same eutectic mixture in 73–97% yield, through a nucleophilic substitution of α-halocarbonyl compounds **71** with NaN_3_. The formation of the pyrazine scaffold is supposed to involve an α-amino ketone intermediate, which is subsequently converted into the corresponding symmetrical 2,5-disubstituted pyrazine **73** (Scheme 24). The DES is suggested to play an important role as a catalytic active species, able to promote an acid-catalyzed formation of the α-amino ketone intermediate from the aryl azide.

Moreover, starting from arylacyl bromide **71** (Ar^1^ = Ph, 4-MeC_6_H_4_, 2,5-(OMe)_2_C_6_H_3_, 2-naphthyl), the authors performed a one-pot two-step azidation/cyclization processes in ChCl/gly DES, obtaining the corresponding pyrazine derivatives in reasonable to excellent yields (64–95%) [66].

## 3. Preparation of *O*- and *S*-Heterocycles in DES Mixtures

### 3.1. Synthesis of Five-Membered O- and S-Heterocycles

**Scheme 25:**
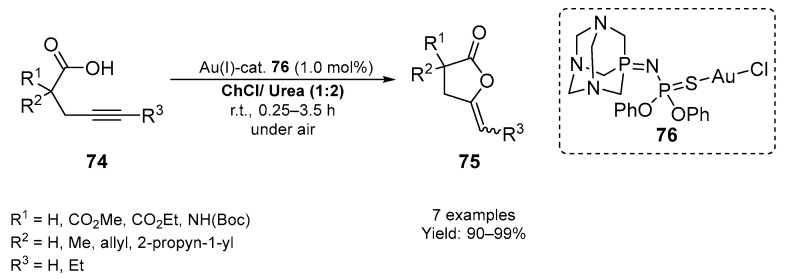
Au(I)-catalyzed cycloisomerization reaction of *γ*-alkynoic acids **74** in ChCl/urea.

In 2014, Rodríguez-Álvarez et al. reported the first example of Au(I)-catalyzed cycloisomerization reaction of various γ-alkynoic acids **74** to form five-membered *O*-heterocycle derivatives dihydrofuran-2-one **75**, in ChCl/urea (1:2) DES, Scheme 25 [69]. The methodology exploits the catalytic activity of neutral Au(I) organometallic complex, containing iminophosphorane ligands, namely [AuCl{κ^1^-*S*-(PTA)=NP(=S)(OPh)_2_}] **76** (PTA = 1,3,5-triaza-7-phosphaadamantane). The desired lactones **75** were afforded an excellent yield (>90%) through standard bench experimental conditions: under air, at room temperature, and without co-catalysts (Scheme 25). Furthermore, the reaction proceeded well with low catalyst loading (1 mol%) and short reaction times (0.25–3.5 h). The authors found that the catalytic complex **76** was much more active in the ChCl/urea eutectic mixture compared to its polyalcohol-based counterparts [ChCl/gly or ChCl/ethylene glycol (EG)]. This could be due to the basic character of the ChCl/urea mixture and its more dipolar nature with respect to the other DESs employed. The polarity of the medium allowed easy and efficient recycling of the catalytic complex and DES for four successive cycles leaving the yield of the product almost unchanged.

**Scheme 26:**
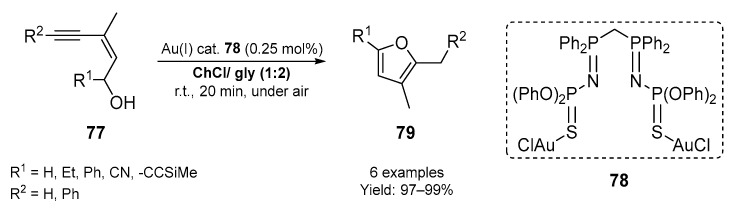
Au(I)-catalyzed cycloisomerization reaction of (*Z*)-enynols **77** in ChCl/gly DES.

A year later, the same research group, developed another cycloisomerization reaction of (*Z*)-enynols **77** to afford furane derivatives **79**, catalyzed by the neutral bis(iminophosphorane) Au(I) complex, namely [Au_2_Cl_2_(μ^2^-*S*,*S*-CH_2_{P(=NP(=S)(OPh)_2_)Ph_2_}_2_)] **78**, in the eutectic mixture ChCl/gly [70]. The synthesis of furane motifs is of great interest due to their presence in a plethora of natural molecules with the most disparate uses (i.e., flavors, fragrances, and drugs) [71,72] and in conducting materials [73]. The methodology proceeds well with a very low catalyst loading (0.25 mol%), under mild reaction conditions (room temperature, under air, and in short reaction time), obtaining the desired furanes **79** in excellent yield (97–99%, Scheme 26). The procedure was also applied to a large-scale reaction (starting from 10 mmol of the substrate) and allowed to recycle the DES-catalyst system for up to ten consecutive cycles, without compromising the process efficiency.

**Scheme 27:**
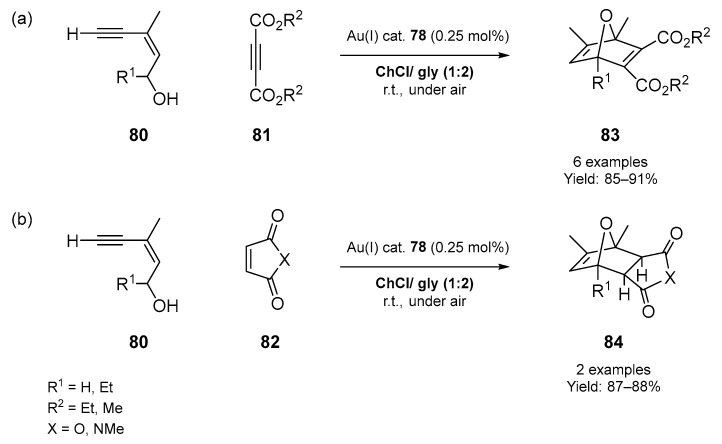
Synthesis of 7-oxanorbornanedienes **83** and *exo*-7-oxanorbornanes **84** in ChCl/gly DES.

Moreover, the same reaction system was employed to carry out the one-pot tandem cycloisomerization/Dies-Alder reaction between (*Z*)-enynols **80** and the activated diethylacetylenedicarboxylate **81** for the synthesis of the *O*-containing heterocycles 7-oxanorbornadienes **83** with excellent yields (85–91%, Scheme 27a). Furthermore, this one-pot tandem methodology was also active when an electron-poor unsaturated substrate **82** (i.e., maleic anhydride and *N*-methylmaleimide) was employed for the synthesis of *exo*-regioisomers 7-oxanorbornanes **84** (Scheme 27b) in a selective manner.

Regarding the class of five-membered *O*-containing heterocycles, dihydroisobenzofuranes are a very widespread molecular structure in a plethora of natural substances [74]. Furthermore, the heterocyclic 1,3-diyhdroisobenzofurane functionality has been demonstrated to have among the most disparate therapeutic activities [75,76,77].

**Scheme 28:**
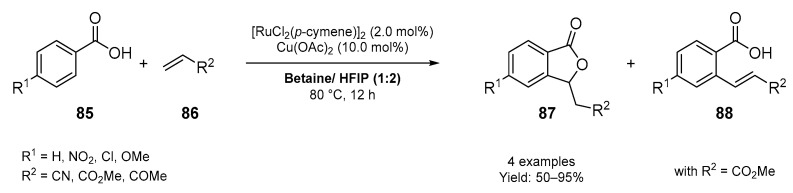
C-H activation, catalyzed by [RuCl_2_(*p*-cymene)]_2_, between benzoic acid derivatives **85** and electron-poor olefins **86** in Betaine/HFIP (1:2) DES.

Very recently, in 2022, Ramón and co-workers developed the C-H activation between benzoic acid derivatives **85** and electron-poor olefins **86**, catalyzed by [RuCl_2_(*p*-cymene)]_2_ for the synthesis of variously substituted 1,3-diyhdrobenzoisobenzofuranes **87** (Scheme 28) [78]. The reaction proceeds well with low catalyst loading (2 mol%) in the eutectic mixture composed of betaine and hexafluoro *i*-propanol (HFIP) (1:2), affording the diyhdroisobenzofurane derivatives in good to excellent yields. Surprisingly, when methyl acrylate was employed as a substrate, a mixture of both cyclic **87** and acyclic **88** products were obtained (Scheme 28).

**Scheme 29:**
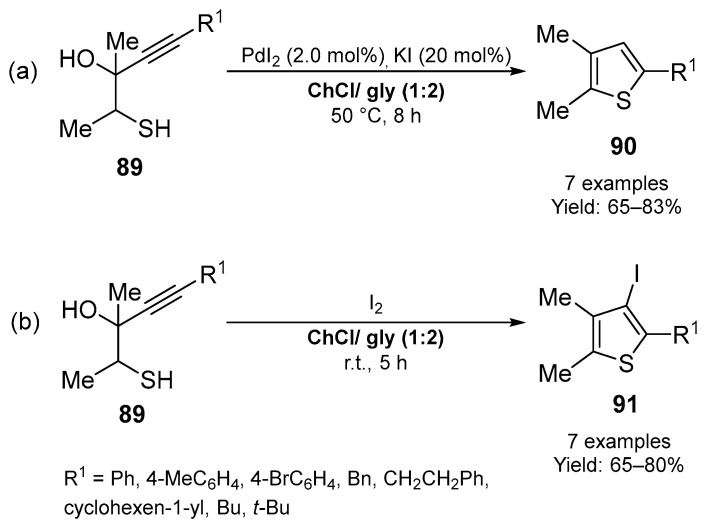
Hetero-cyclodehydration and iodocyclization of 1-mercapto-3-yn-2-ols **89** in ChCl/gly.

Moreover, also the synthesis of the thiophen ring has been achieved in DES mixtures. Particularly, in 2016, Mancuso and coworkers performed, in ChCl/gly as a green solvent, a hetero-cyclodehydration reaction of 1-mercapto-3-yn-2-ols **89** to obtain substituted thiophens **90** [79]. The process was carried out for 8 h at 50 °C in the presence of a PdI_2_/KI catalytic system, leading to the desired heterocycles **90** in 65–83% yields (Scheme 29a). For all the examples reported, the DES/catalytic system could be successfully recycled up to six runs. Furthermore, the same substrates **89** underwent an iodocyclization reaction, performed at room temperature for 5 h with I_2_, affording 3-iodothiophene derivatives **91** in moderate to good yields (62–80%, Scheme 29b). Furthermore, in the case of the iodocyclization process, the eutectic mixture ChCl/gly could be easily reused several times (up to six runs), without any appreciable decrease in the product yield [79].

**Scheme 30:**
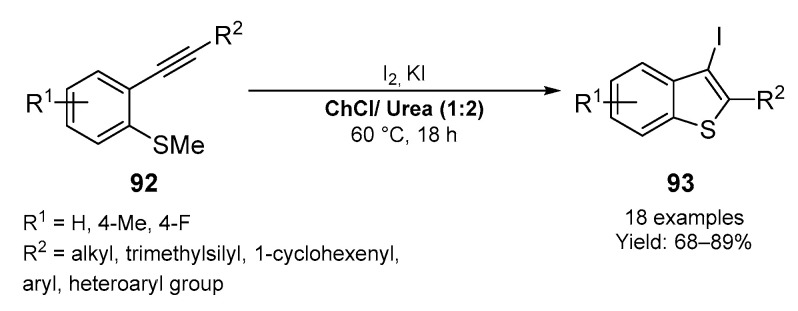
Iodocyclization reaction of 2-methylthiophenylacetylenes (**92**) in DES.

More recently, the same authors reported, in the DES ChCl/urea, an iodocyclization reaction of 2-methylthiophenylacetylenes (**92**) to produce 3-iodobenzothiophenes (**93**) [80], a useful precursor of bio-active compounds (Scheme 30) [81]. The reaction was carried out at 60 °C for 18 h with differently substituted substrates, in the presence of I_2_ and KI, which probably supports sulfur demethylation. The possibility to recycle the DES solvent was also assessed, showing no appreciable yield loss for six consecutive runs. Moreover, the synthesized heterocycles **93** were further employed as cross-coupling partners in Sonogashira and Suzuki reactions to obtain functionalized benzothiophene derivatives [81].

### 3.2. Synthesis of Six-Membered O-Heterocycles

Coumarin backbone has attracted great interest from both synthetic and medicinal chemists because of its photochemical properties [82] as well as its wide range of biological activities including anti-HIV [83] and anticancer properties [84].

**Scheme 31:**
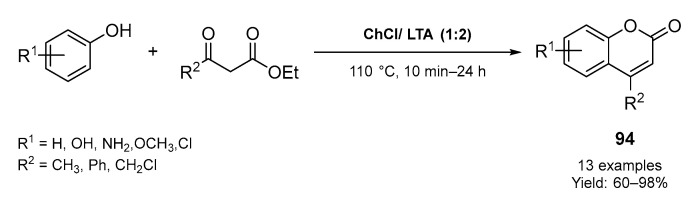
Synthesis of functionalized coumarins **94** in ChCl/LTADES.

In 2022, Rather and Ali reported an eco-friendly Pechmann condensation for the synthesis of functionalized coumarins [85]. The reaction, performed at 110 °C in the green medium ChCl/LTA, afforded variously substituted coumarins **94** in good to excellent yields (60–98%), starting from various phenols and β-ketoesters (Scheme 31). Depending on the stereo-electronic properties of the reactants, the reaction time was observed to change considerably: the best result was achieved with the use of phloroglucinol as phenol and ethyl acetoacetate as β-ketoester; indeed, the desired Pechmann condensation product was obtained in just 10 min with a 98% of yield [85].

**Scheme 32:**
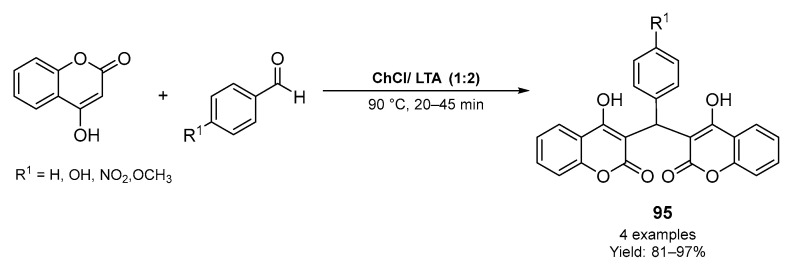
Synthesis of bis-coumarins **95** in ChCl/LTADES.

Moreover, the same eutectic mixture ChCl/LTA at a lower reaction temperature (90 °C) was employed to prepare bis-coumarins **95** in 81–97% yields, by reacting 7-hydroxycoumarin with aromatic aldehydes (Scheme 32). A dual role of the DES as the solvent as well as the catalyst, via hydrogen bonding interactions, was suggested; furthermore, the eutectic mixture was effectively recycled for four runs and no significant decrease in the product yield was observed [85].

**Scheme 33:**
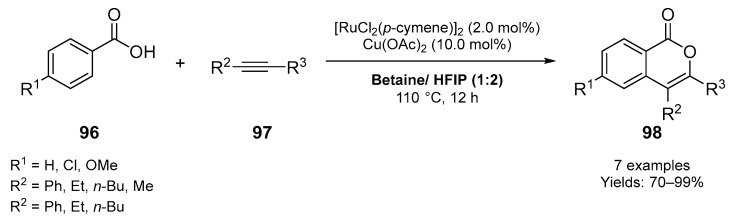
C-H activation reaction for the synthesis of isocumarines **98** in Betaine/HFIP DES.

In the same year, González-Gallardo et al. reported a methodology to produce isocumarine derivatives via a C-H activation cross-coupling reaction between benzoic acids **96** and disubstituted alkynes **97** [78]. Isocumarines scaffolds are a fascinating class of natural products with synthetic and pharmaceutical applications [86]. The reaction was catalyzed by the commercially available [RuCl_2_(*p*-cymene)]_2_ pre-catalyst in the Betaine/HFIP (1:2) eutectic solvent, using Cu(OAc)_2_ as an oxidant. The methodology tolerated various internal alkynes with aromatic, aryl-alkyl, and alkyl-alkyl substituents affording the desired isocumarine derivatives **98** in good to excellent yields (70–99%, Scheme 33).

As described by the authors, the same catalytic system was also effective for the C-H activation of variously decorated *N*-metoxybenzamides (used instead of benzoic acid derivatives) and internal alkynes to afford isoquinoline derivatives in poor to excellent yields (20–93%).

Another important *O*-containing heterocycle, with the most diverse technological and pharmacological activities [87,88], is the xanthane ring. Moreover, this heterocycle has a high synthetic value because of its intrinsic reactivity related to the inbuilt pyran ring [89].

**Scheme 34:**
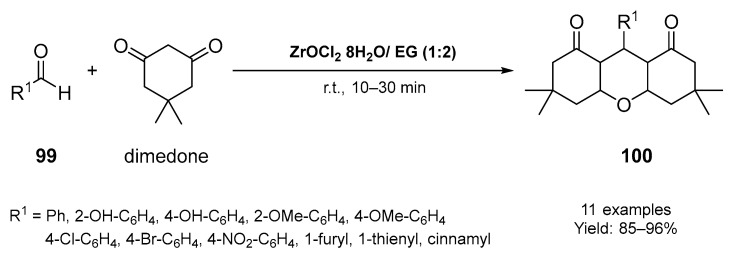
Synthesis of 1,8-dioxooctahydroxanthenes **100** in type IV ZrOCl_2_∙8H_2_O/EG mixture.

In 2021, Shaibuna et al. developed the sustainable synthesis of 1,8-dioxooctahydroxanthenes **100**, through the reaction of aldehydes **99** with dimedone using the type IV DES ZrOCl_2_∙8H_2_O/EG (1:2) as both catalyst and reaction medium [90]. The optimized methodology allowed us to obtain the desired octahydroxanthane derivatives **100** in good to excellent yields (85–96%), in short reaction times (10–30 min), and at room temperature (Scheme 34). The eutectic mixture plays a key role in the reaction by activating the aldehyde group through a hydrogen bond interaction between the EG of the DES and the carbonyl oxygen [90].

**Scheme 35:**
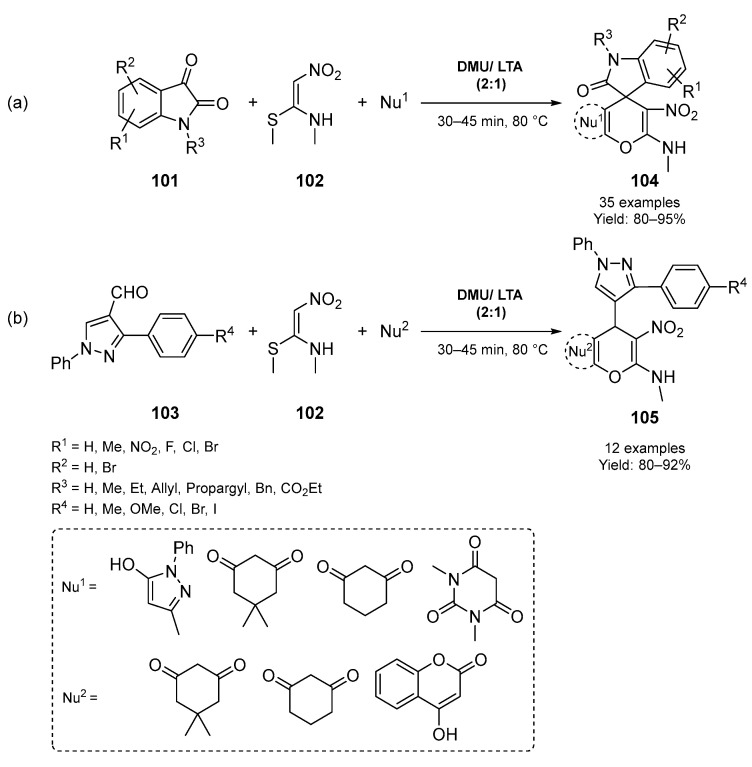
Synthesis of spirochromenes **104** and chromene derivatives **105** in DMU/LTA DES.

Chromenes and spirochromenes are important six-membered *O*-containing heterocycles, with different pharmacological activities [91]. Recently, in 2021, Sathish et al. reported the synthesis of spirochromenes **104** and chromene derivatives **105**, in good yields, using DMU/LTA (2:1) eutectic mixture as a reaction medium, at 80 °C for 30–45 min [92]. The methodology afforded the desired spirochromenes **104** in yields up to 95%, via a three-component reaction involving a nucleophile (i.e., pyrazole, dimedone, 1,3-cyclohexanedione, and *N*,*N*-dimethyl barbituric acid), the isatins derivatives **101** and the nitroolefin derivative **102** (Scheme 35a). Moreover, simply by switching isatines **101** with the pyrazole aldehyde **103**, the same experimental conditions led to the chromene derivatives **105**, in excellent yields (80–92%, Scheme 35b) [92].

## 4. Miscellaneous

The development of synthetic protocols for the preparation of multi-heterocyclic compounds has attracted researchers in the last few years due to their structural complexity and variety. This type of heterocycles has demonstrated their importance in coordination chemistry, material sciences, and the pharmaceutical industry [93].

**Scheme 36:**
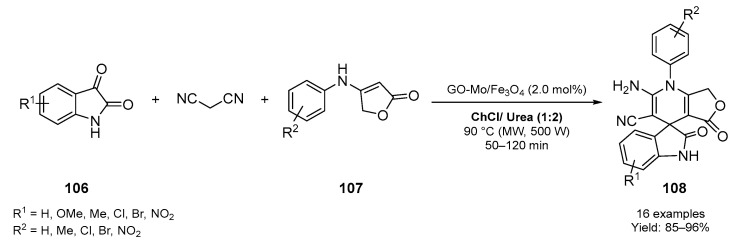
Synthesis of spirooxindole dihydropyridines derivatives **108** via a three-component reaction catalyzed by GO-Mo/Fe_3_O_4_ in ChCl/urea DES.

Indeed, Zhang and co-workers developed a three-component reaction between isatins **106**, malonitrile, and anilinolactones **107**, mediated by supported molybdenum on graphene oxide (GO-Mo)/Fe_3_O_4_ as the heterogeneous magnetic catalyst, for the synthesis of spirooxindole dihydropyridines **108** in ChCl/urea solvent [94]. The reaction proceeded well with low catalyst loading (2 mol%), and required microwave irradiation at 500 W to be conducted in short reaction times (50–120 min). The methodology demonstrated a wide substrate scope and proceeded smoothly with various decorated isatins **106** and anilinolactones **107**, affording the desired spirooxindole dihydropyridines derivatives **108** in good to excellent yields (Scheme 36) [94]. Moreover, the catalyst was easily recovered, thanks to its magnetic characteristics, and effectively reused with the DES for eight subsequent cycles, leaving unchanged the process efficiency.

**Scheme 37:**
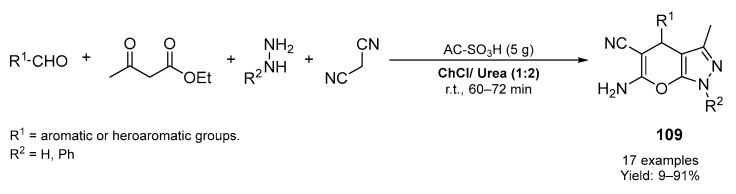
AC-SO_3_H-catalyzed synthesis of functionalized dihydropyrano[2,3-*c*]pyrazoles **109** in DES.

In 2021, the eutectic mixture ChCl/urea was used as an eco-friendly solvent by Nguyen and coworkers to carry out a four-component reaction involving an aromatic or heteroaromatic aldehyde, ethyl acetoacetate, hydrazine (hydrazine hydrate or phenylhydrazine), and malononitrile (Scheme 37) [95]. This reaction was applied to the preparation of pyranopyrazoles **109**, scaffolds with interesting biological properties [96], and employed a synthesized sulfonated amorphous carbon (AC-SO_3_H) as the catalyst at room temperature (Scheme 37). The pyranopyrazole derivatives **109** were obtained in a range of yields from 9% to 91%. Particularly, steric hindrances of the aldehyde had a significant influence on the yield process, and the efficiency of forming the desired heterocycle **109** was the lowest (9% yield) when the reaction was performed with benzaldehyde-containing substituents at the ortho position (e.g., OH). On the contrary, the desired product was obtained with excellent yield by using heterocyclic aldehydes, with the best results for 5-chlorofuran-2-carbaldehyde (product yield: 91%) [95]. Although the use of catalytic system AC-SO_3_H/[ChCl][urea]_2_ provided the desired heterocycles in lower yield than other procedures drawn from the literature, this methodology showed some advantages such as eco-friendly, simple procedure, and mild reaction conditions.

**Scheme 38:**
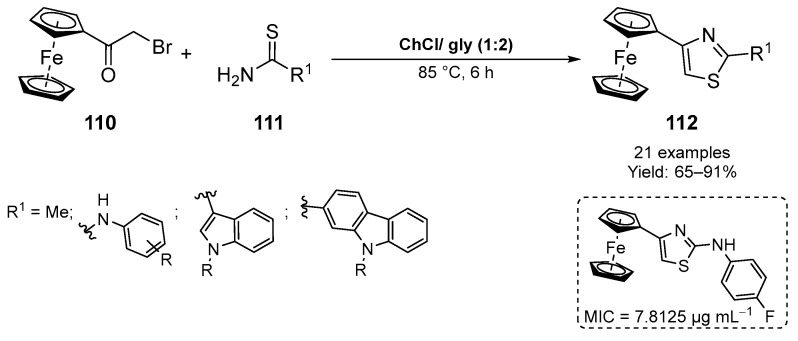
Preparation of ferrocene-based thiazole derivatives **112** in ChCl/gly eutectic solvent.

Other heterocycles with biological activities were recently prepared by Zhao et al. [97]. Specifically, they performed a green Hantzsch synthesis of ferrocene–thiazole hybrids **112** in the DES ChCl/gly at 85 °C, starting from bromoacetylferrocene **110** and variously substituted aryl thioureas or carbothioamides **111**. This synthetic route led to heterocycles **112** in good to excellent yields (65–91%), Scheme 38. The ChCl-based eutectic mixture acts both as an eco-friendly medium and catalyst via hydrogen bond interactions, and it can be reused for up to three consecutive runs without any appreciable reduction in the product yield. Furthermore, a preliminary in vitro antibacterial assay of the synthesized molecules **112** showed significant antibacterial activity against Gram (+) *B. subtilis* and Gram (−) *E. coli* of the fluoro-substituted ferrocene–thiazole hybrid obtained from compound **110** and 1-(4-fluorophenyl)thiourea [MIC (minimum inhibitory concentration) value of 7.8125 µg mL^−1^, Scheme 38] [97].

## 5. Conclusions

After reviewing the syntheses of *N*-, *O*-, and *S*-heterocycles in eutectic mixtures, several general observations can be made. The experimental conditions described are generally mild and the majority of authors reported the use of neutral reactants. Additionally, the non-volatile nature of DESs and their compatibility with water enables the recycling of both metal catalysts and the solvent, ultimately reducing costs and waste production. While the efficacy of these synthetic methodologies is indisputable, the role of DES, which is believed to promote H-bond catalysis, requires further investigation to validate its supposed unique activities that cannot be explained solely through the general principles of solvent effects.

## Data Availability

Not applicable.
